# JNK‐IN‐8, a c‐Jun N‐terminal kinase inhibitor, improves functional recovery through suppressing neuroinflammation in ischemic stroke

**DOI:** 10.1002/jcp.29183

**Published:** 2019-09-20

**Authors:** Jianjian Zheng, Qinxue Dai, Kunyuan Han, Wandong Hong, Danyun Jia, Yunchang Mo, Ya Lv, Hongli Tang, Hongxing Fu, Wujun Geng

**Affiliations:** ^1^ Key Laboratory of Diagnosis and Treatment of Severe Hepato‐Pancreatic Diseases The First Affiliated Hospital of Wenzhou Medical University Wenzhou Zhejiang People's Republic of China; ^2^ Department of Anesthesiology The First Affiliated Hospital of Wenzhou Medical University Wenzhou Zhejiang People's Republic of China; ^3^ Department of Gastroenterology and Hepatology The First Affiliated Hospital of Wenzhou Medical University Wenzhou Zhejiang People's Republic of China; ^4^ School of Pharmaceutical Wenzhou Medical University Wenzhou Zhejiang People's Republic of China

**Keywords:** ischemia brain injury, JNK, JNK‐IN‐8, neuroinflammation, NF‐κB

## Abstract

C‐Jun N‐terminal kinase (JNK) is a pivotal MAPK (mitogen‐activated protein kinase), which activated by ischemia brain injury and plays a fairly crucial function in cerebral ischemic injury. Emerging studies demonstrated that JNK‐IN‐8 (a JNK inhibitor with high specificity) regulates traumatic brain injury through controlling neuronal apoptosis and inflammation. However, the function of JNK‐IN‐8 in ischemic stroke and the mechanisms underlying of JNK‐IN‐8 about neuroprotection are not well understood. In this work, male rats were treated with JNK‐IN‐8 after transient middle cerebral artery occlusion, and then the modified improved neurological function score (mNSS), the foot‐fault test (FFT), interleukin‐1β (IL‐1β), IL‐6, and tumor necrosis factor‐α (TNF‐α) levels were assessed. We found that JNK‐IN‐8‐treated rats with MCAO exerted an observable melioration in space learning as tested by the improved mNSS, and showed sensorimotor functional recovery as measured by the FFT. JNK‐IN‐8 also played anti‐inflammatory roles as indicated through decreased activation of microglia and decreased IL‐6, IL‐1β, and TNF‐α expression. Furthermore, JNK‐IN‐8 suppressed the activation of JNK and nuclear factor‐κB (NF‐κB) signaling as indicated by the decreased level of phosphorylated‐JNK and p65. All data demonstrate that JNK‐IN‐8 inhibits neuroinflammation and improved neurological function by inhibiting JNK/NF‐κB and is a promising agent for the prevention of ischemic brain injury.

## Graphical abstract

All data demonstrate that c‐Jun N‐terminal kinase (JNK)‐IN‐8 inhibits neuroinflammation and improved neurological function by inhibiting JNK/nuclear factor‐κB (NF‐κB) and is a promising agent for the prevention of ischemic brain injury.

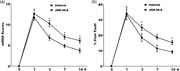

## 1 INTRODUCTION

Ischemic stroke remains the main types of stroke and leads to long‐term disability and countless efforts have been made towards the therapeutic treatment on the disease. Despite current diagnosis and prognosis for ischemic stroke can be facilitated by genetic or transcriptomic biomarkers, it becomes seriously limited in terms of early stroke management (Amouyel, 2012). It has been demonstrated widely that neuroinflammation acts as a fairly crucial function on the pathophysiology of ischemic injury stroke in which local neuroinflammation can cause neuronal damage and degeneration (Jin, Liu, Zhang, Nanda, & Li, 2013). Clinical research indicated that prognoses of stroke can be significantly influenced by systemic inflammation (Emsley & Hopkins, 2008) while inhibition on inflammatory responses could decrease brain injury (Yilmaz & Granger, 2008). Therefore, a comprehensive understanding of regulation on inflammatory processes in response to the brain insult can be a precondition of developing an effective treatment for ischemic stroke.

C‐Jun N‐terminal kinase (JNK), a pivotal mitogen‐activated protein kinase (MAPK), which relates to inflammatory processes in many diseases (Chen & Tan, 2000; Choudhury, Ghosh, Gupta, Mukherjee, & Chattopadhyay, 2015; Utsugi et al., 2003; Wardyn, Ponsford, & Sanderson, 2015). JNK is considered as a major stress‐responsive kinase and JNK signaling is reportedly associated with neuroinflammation, blood‐brain barrier disruption, and oligodendroglia apoptosis in brain injury (Manning & Davis, 2003). Studies on novel therapeutic targets neuroinflammation and neuropathic pain have considered JNK as a promising candidate due to its regulatory roles in inducing neuroinflammation in vivo and in vitro (Ramesh, 2014). A specific JNK inhibitor, JNK‐IN‐8, has been used to inquiry JNK precise effect in many pathways and diseases (Zhang et al., 2012). Previous research has shown that a dual NO‐donating oxime and JNK inhibitor is reported to safeguard cells against cerebral ischemia‐reperfusion injury, indicating JNK inhibitor use is accessible for investigation on functions of JNK in ischemia injury (Atochin et al., 2016). Specifically, JNK‐IN‐8 is reported to significantly suppress tumor growth in vitro and in vivo, directly proving JNK regulating breast cancer tumorigenesis (Xie et al., 2017). These studies exhibit the valuable aspect of JNK inhibitors in protecting against molecular occurrence of different diseases, however, the precise effect of JNK‐IN‐8 on neuroinflammation related to ischemic stroke remains unclear.

In this study, our purpose is to explore whether JNK‐IN‐8 can improve functional recovery through suppressing neuroinflammation in ischemic stroke. We used established transient middle cerebral artery occlusion (tMCAO) rat model by JNK‐IN‐8 treatment. We assessed the modified improved neurological function score (mNSS), the foot‐fault test (FFT), the interleukin‐1β (IL‐1β), IL‐6, and tumor necrosis factor‐α (TNF‐α) level. Current data demonstrated that JNK‐IN‐8 exerted a neuroprotective and anti‐inflammatory effect and suppressed JNK activation and nuclear factor‐κB (NF‐κB) signaling activation in ischemic brain injury, proposing a potent treatment for ischemic brain injury.

## 2 MATERIALS AND METHODS

### 2.1 MCAO model

Animal experiments were confirmed at the Committee for Animal Experiments at Wenzhou Medical University. Adult male Sprague–Dawley (SD) rats (6–8 weeks of age, 200–250 g) were obtained from the Experimental Animal Center of Wenzhou Medical University (China) and maintained in a specific sterile environment for the duration of the current study. Animals were randomized to the sham‐operated group (*n* = 5) and experimental group (*n* = 15), which was then subdivided into two subgroups (vehicle and JNK‐IN‐8 group).

The animals were subjected to MCAO according to the previous description (Hata et al., 1998; Yu et al., 2018). In Brief, each rat was anesthetized by choral hydrate (350 mg/kg) intraperitoneally. The right vessels, including the common carotid artery, external carotid artery (ECA), and internal carotid artery (ICA) were exposed by a midline cervical incision. The ECA was coagulated and inserted into the ICA through the ECA to occlude the MCA. And 2 hrs later, the suture was withdrawn to allow MCA perfusion. The blood flow of MCA was observed to verify the occurrence of ischemia by a Laser Doppler flowmetry (Oxford Optronix, UK). SD rats of the sham‐operated group were dealt with the same procedures except for MCA occlusion. A heating pad (Malvern, UK) was used to keep the temperature at 37.0°C and the rats were kept on it until the closure of the skin incision.

### 2.2 Cell culture

The murine BV2 microglial cells, collected from the ATCC (Manassas, VA), treated with Dulbecco's modified Eagle's medium (DMEM) contained with 10% Fetal bovine serum and 1% penicillin as well as streptomycin (YBio, Shanghai, China), and were were incubated at 37°C in a 5% CO_2_ incubator. The oxygen‐glucose deprivation was carried out through exposure of BV2 cells to DMEM containing no glucose or serum in a specific environment (5% CO_2_ and 95% nitrogen) for 6 hr.

### 2.3 Drugs

Rats were subjected to MCAO, and then the vehicle (150 μl saline as well as 20% dimethyl sulfoxide [DMSO] in phosphate‐buffered saline) or JNK‐IN‐8 (Selleck Chemicals, TX; 20 mg/kg in 20% DMSO) was injected into rats by intraperitoneal injection. BV2 microglial cells were cultured in JNK‐IN‐8 (10 mM) and then TNF‐a, IL‐1β, and IL‐6 levels were assessed using quantitative polymerase chain reaction (qPCR) and enzyme‐linked immunosorbent assay (ELISA).

### 2.4 Behavioral tests

The mNSS test was carried out to assess neurological function according to the previous description (Chen et al., 2001). This experiment was conducted before the rats received the injury and at 1, 3, 7, and 14 days after MCAO. About the mNSS, neurological functions including motor (muscle status and abnormal movement), sensory systems (visual, tactile, and proprioceptive) and reflexes. The mNSS was graded on a scale of 0–18, and the higher score indicates a more severe injury.

The sensorimotor function was assessed by the FFT according to the previous description (Zhang et al., 2015). The rats were placed on an elevated grid to walk, and the number of foot‐fault errors (the paw slips between the wires) was recorded.

### 2.5 Quantitative real‐time polymerase chain reaction

The total RNA was extracted from microglia with TRIzol reagent (Ambion) as the manufacturer's manuals. Reverse transcription (RT) was performed by Prime Script^TM^ Master Mix and oligo‐dT primers (Generay, Shanghai, China). qPCR was carried out by 2 × SYBR Green Mix (Vazyme Biotech, China) on an Applied Biosystems 7500 fast instrument (Applied Biosystems, CA). β‐Actin were designed as an internal standard for messenger RNA. The primer information was shown in Table S1.

### 2.6 Western blotting

Total protein was isolated from cerebral cortex or microglia with homogenization in lysis buffer and centrifuged at 12,000 rpm about 15 min. The BCA kit (Cell Signaling Technology, Boston, MA) was used to determine the protein concentrations. The western blotting was conducted according to the previous description (Zhu et al., 2019). Then, the proteins were incubated overnight at 4℃ with specific primary antibodies, which including Iba‐1 (1:1,000; ab178846; Abcam), JNK (1:1,000; #9252; Cell Signaling Technology, MA), phosphorylated‐JNK (p‐JNK; 1:1,000; #9251; Cell Signaling Technology), P65 (1:1,000; #3039; Cell Signaling Technology), I‐kBa (1:1,000; #9242; Cell Signaling Technology), and subsequently detected using the secondary antibody (1:2,000; Cell Signaling Technology).

### 2.7 ELISA assay for inflammatory cytokines

The ELISA assay was performed using ELISA kits (Biosource International Inc) as instructions. Briefly, to quantify TNF‐α, IL‐1β, as well as IL‐6 protein level in the tissue of the brain, 96‐well plates coated with indicated antibodies, were treated with the addition of the supernatant of brain tissue homogenate (1:20 dilution). After the reaction between enzyme and substrate, the absorbances of the sample was assessed at 450 nm using a microplate reader. All the procedures were repeated for at least three times.

### 2.8 Immunofluorescence staining

The immunofluorescence staining in brain tissues was carried out according to the previous description (Burton, Sparkman, & Johnson, 2011). Specific primary antibody against Iba‐1 (1:1,500; ab178846) was used to mark the section at 4°C overnight. Anti‐rat Alexa Fluor 488 (1:1,000; Invitrogen) as secondary antibody was added to incubate the section and then counterstained with 4′,6‐diamidino‐2‐phenylindole (ATOM). The samples were observed and analyzed by the LEICA TCS SPE microscope (Leica, Germany) and LEICA software LAS AF, respectively. And the positive cells were statistically counted and plotted.

### 2.9 Statistics

All data were expressed as mean ± standard deviation. Student's *t* test was used to assess differences between groups. Values less than .05 was defined as the criteria to determine significance.

## 3 RESULTS

### 3.1 JNK‐IN‐8 enhances functional recovery after stroke

To test the effect of JNK‐IN‐8 on regulating cerebral ischemic injury, the sensorimotor performance of the stroke severity was evaluated by assaying foot‐fault and mNSS after JNK‐IN‐8 treatment. Rats received vehicle or JNK‐IN‐8 intraperitoneally at 2 hr after MCAO. The mNSS and FFT were carried out before the treatment after MCAO, at Day 1, 3, 7, and 14 after MCAO.

**Figure 1:**
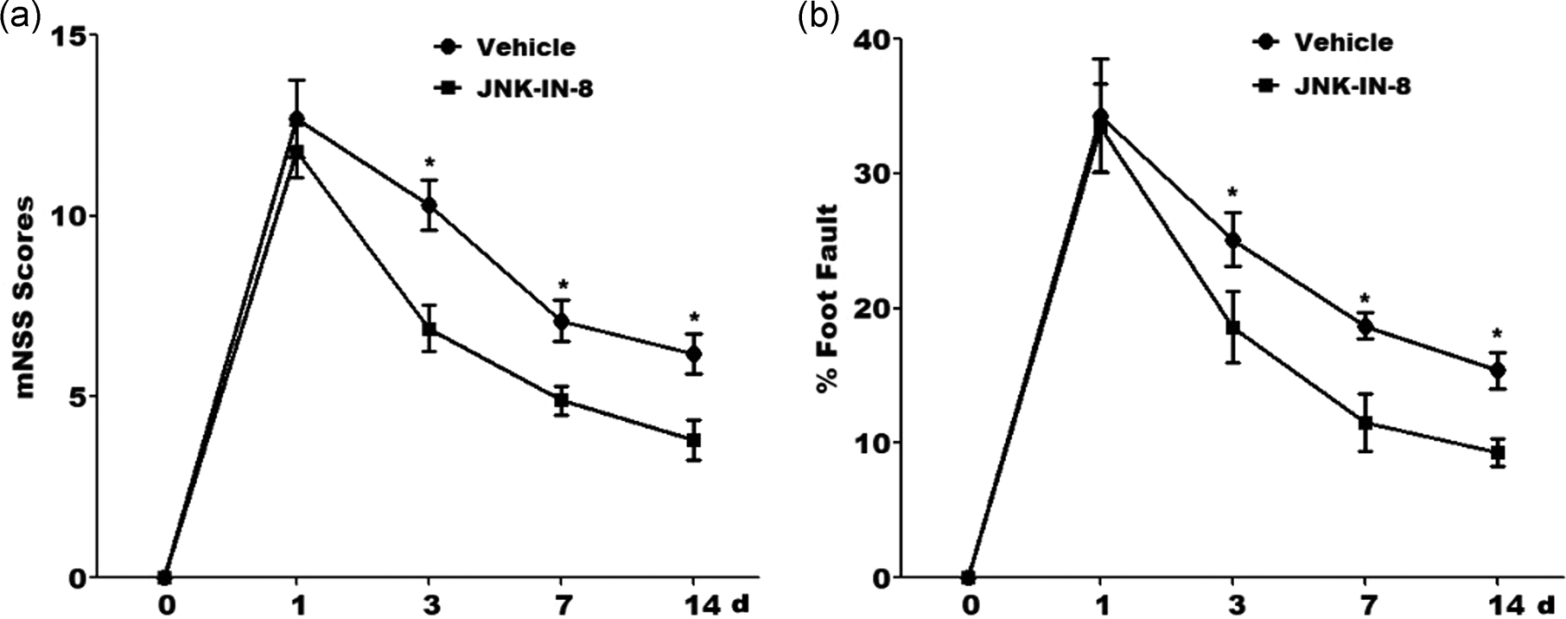
JNK‐IN‐8 improves functional recovery after stroke. (a) mNSS of rat behavior after MCAO and intraperitoneal injection of vehicle or JNK‐IN‐8 (20 mg/kg). *n* = 5, *p< .05. (b) A foot‐fault test after MCAO and intraperitoneal injection of vehicle or JNK‐IN‐8 (20 mg/kg). *n* = 5, *p < .05. *Single asterisk indicated the significant difference when *p* < .05. JNK, c‐Jun N‐terminal kinase; MCAO, middle cerebral artery occlusion; mNSS, modified improved neurological function score

The mNSS was nearly 12 in rats with MCAO (the vehicle group and JNK‐IN‐8 group) on the first day after MCAO, suggesting that there did not have an observable difference about neurological functional deficits between the two groups before treatment (Figure 1a). Significant decrease of mNSS was discovered over time in vehicle group on Day 3, 7, and 14 after MCAO. Importantly, JNK‐IN‐8 treatment resulted in a significant functional recovery on Day 3, 7, and 14 after MCAO compared with the vehicle (Figure 1a). Compared with the vehicle group, JNK‐IN‐8 treatment decreased the occurrence of the frequency of forelimb and foot faults likewise (Figure 1b). These results demonstrated that JNK‐IN‐8 improves functional recovery after stroke.

### 3.2 JNK‐IN‐8 inhibits microglia activation in vivo after stroke

**Figure 2:**
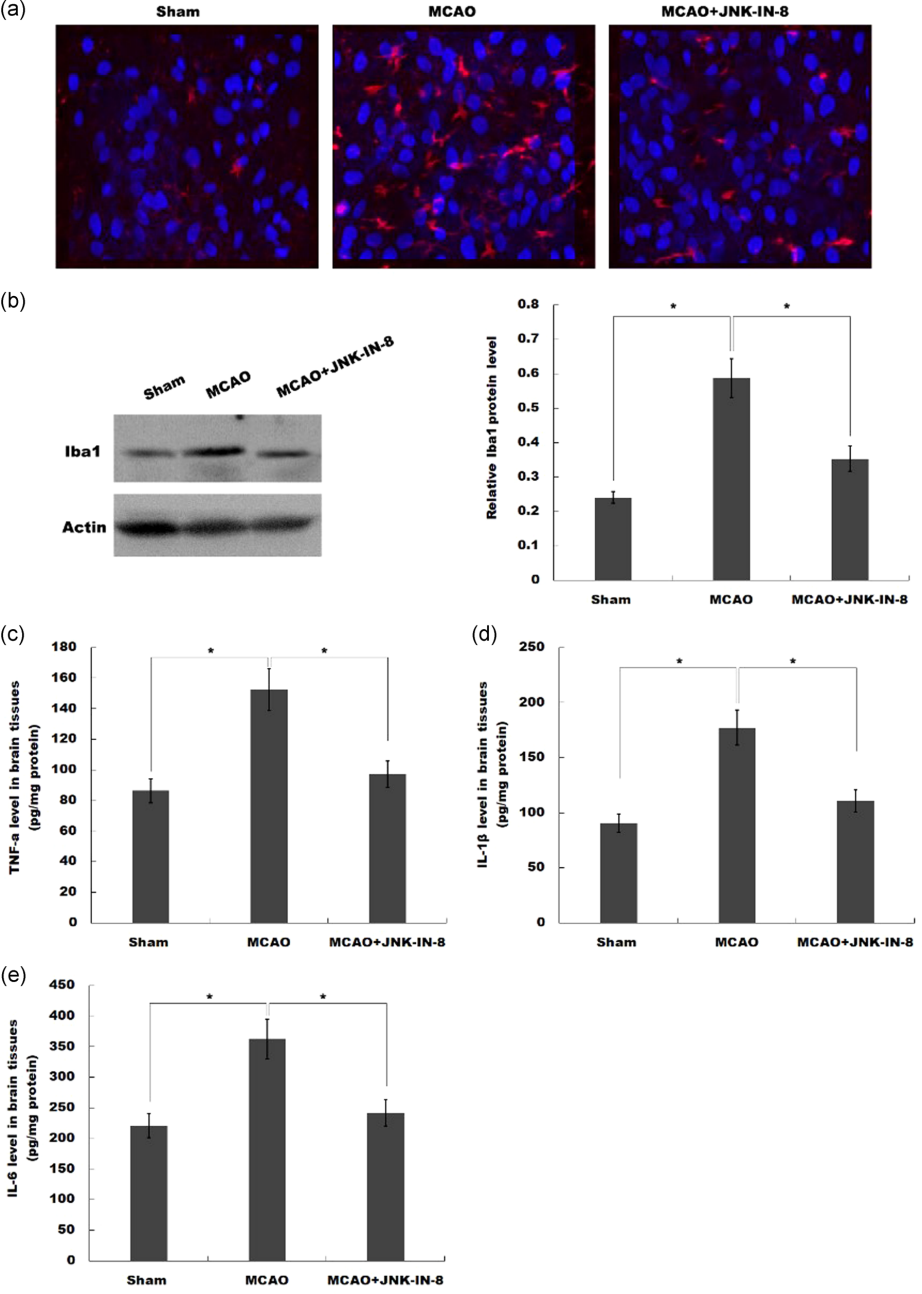
JNK‐IN‐8 inhibits microglia activation in vivo after stroke. (a) Immunofluorescence of microglia cells in the ipsilateral cortex after MCAO and intraperitoneal injection of vehicle or JNK‐IN‐8. (b) The protein expression level of Iba‐1 in the ipsilateral cortex after MCAO and intraperitoneal injection of vehicle or JNK‐IN‐8 was detected using Western blot analysis. (c–e) ELISA analysis of (c) TNF‐α, (d) IL‐1β, and (e) IL‐6 level in brain tissues after MCAO and intraperitoneal injection of vehicle or JNK‐IN‐8. MCAO, middle cerebral artery occlusion; ELISA, enzyme‐linked immunosorbent assay; IL, interleukin; JNK, c‐Jun N‐terminal kinase; TNF‐α, tumor necrosis factor‐α. *p < .05 [Color figure can be viewed at wileyonlinelibrary.com]

To unveil the potential mechanism of JNK‐IN‐8 on treating MCAO rats, microglia activation was analyzed by immunofluorescence analysis using Iba‐1 antibody. Figure 2a showed that MCAO resulted in a significant microglial activation in ipsilateral cortex at 4 hr after MCAO as indicated through the intensive ramified Iba‐1‐positive staining, which was significantly inhibited after JNK‐IN‐8 treatment. To verify JNK‐IN‐8 effect on microglial activation, the expression level of Iba‐1 protein in the ipsilateral cortex was assayed through western blot. Compared with the control group of rats, the protein level of Iba‐1 was obviously added in the ipsilateral cortex of MCAO rats, whereas this increase was inhibited by JNK‐IN‐8 treatment (Figure 2b).

We then assessed JNK‐IN‐8 effects on the production of proinflammatory cytokines in brain tissues. ELISA analysis was carried out to assay TNF‐α, IL‐1β as well as IL‐6 protein level in cerebral tissues. MCAO resulted in an obvious increase of TNF‐α, IL‐1β, and IL‐6, whereas JNK‐IN‐8 treatment inhibited these proinflammatory cytokines production. These data suggested that JNK‐IN‐8 plays important roles in inhibiting cerebral ischemia‐induced microglial activation and subsequent neuroinflammation (Figure 2c–e).

### 3.3 JNK‐IN‐8 inhibits the activation of JNK and NK‐κB signaling

**Figure 3:**
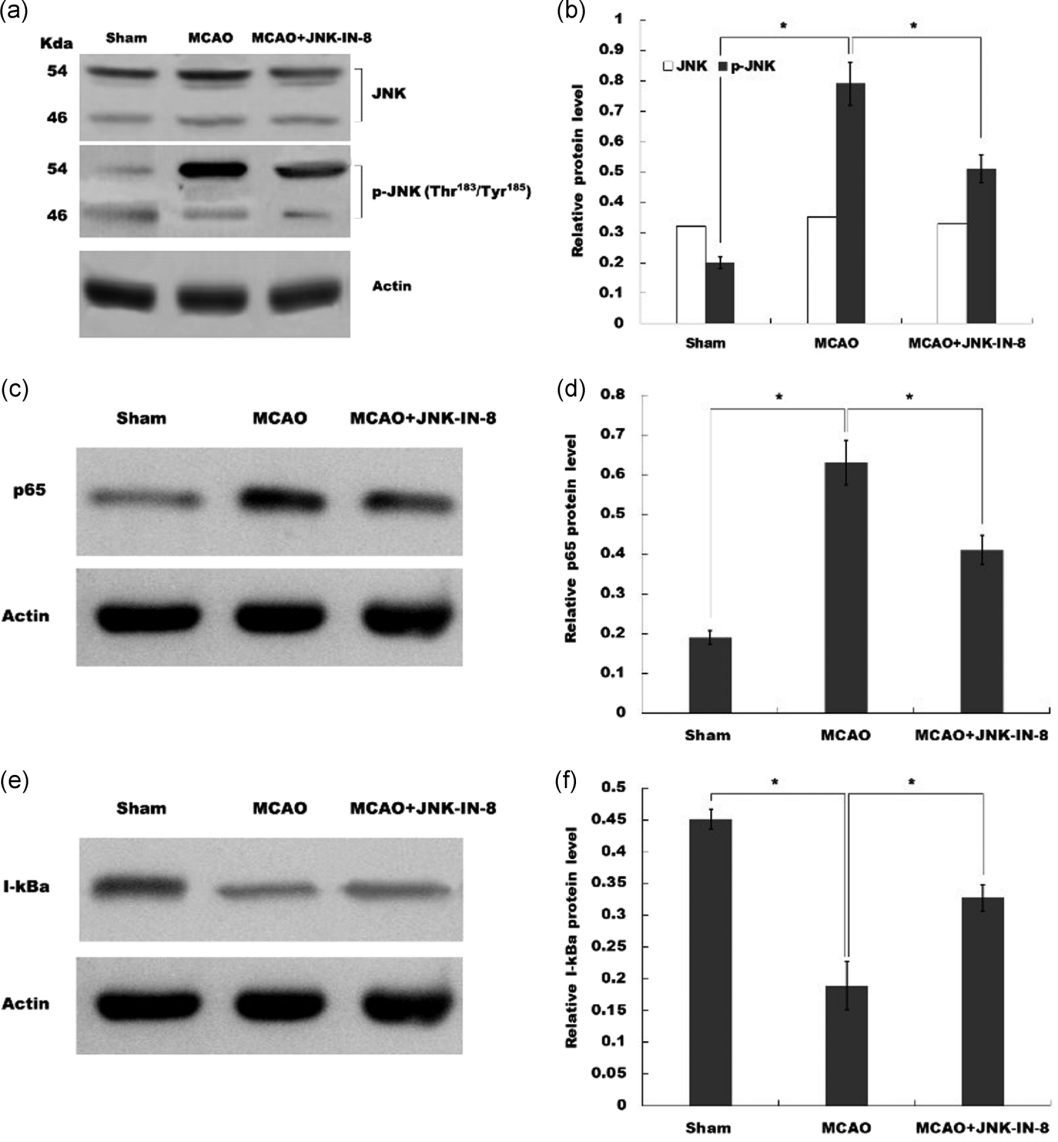
JNK‐IN‐8 inhibits the activation of JNK‐NK‐κB pathway. (a and b) Western blot detection of brain JNK and p‐JNK levels after MCAO and intraperitoneal injection of vehicle or JNK‐IN‐8. (c and d) Western blot analysis of brain p65 levels after MCAO and intraperitoneal injection of vehicle or JNK‐IN‐8. (e and f) Western blot analysis of brain I‐kBa levels after MCAO and intraperitoneal injection of vehicle or JNK‐IN‐8. MCAO, middle cerebral artery occlusion; p‐JNK, phosphorylated c‐Jun N‐terminal kinase. *p < .05

We then investigated whether JNK‐IN‐8 treatment inhibited JNK activation in vivo after MCAO. Western Blot indicated that brain p‐JNK levels were increased at 4 hr after MCAO, and JNK‐IN‐8 treatment significantly attenuated brain p‐JNK level compared with the vehicle; indicating JNK pathway was activated after MCAO and ischemia‐induced JNK activation were inhibited after JNK‐IN‐8 treatment (Figure 3a,b).

Previous research has shown that JNK‐IN‐8 possesses the potential to inhibit NF‐κB activation in breast carcinoma cells (Ebelt et al., 2017), and NF‐κB is a pivotal upstream modulator for proinflammatory cytokines in microglia (Jiang et al., 2017; Simmons et al., 2016). Therefore, we further investigated whether the neuroinflammation‐relieving effect of JNK‐IN‐8 in MCAO rats is related to NF‐κB activation. Figure 3c–f showed that the p65 expression was increased and I‐κBα level was reduced by ischemic injury, indicating that NF‐κB signaling was activated after MCAO, and the change in NF‐κB signaling was reversed by JNK‐IN‐8 treatment.

### 3.4 JNK‐IN‐8 inhibits microglia activation and the production of proinflammatory cytokines in vitro

**Figure 4:**
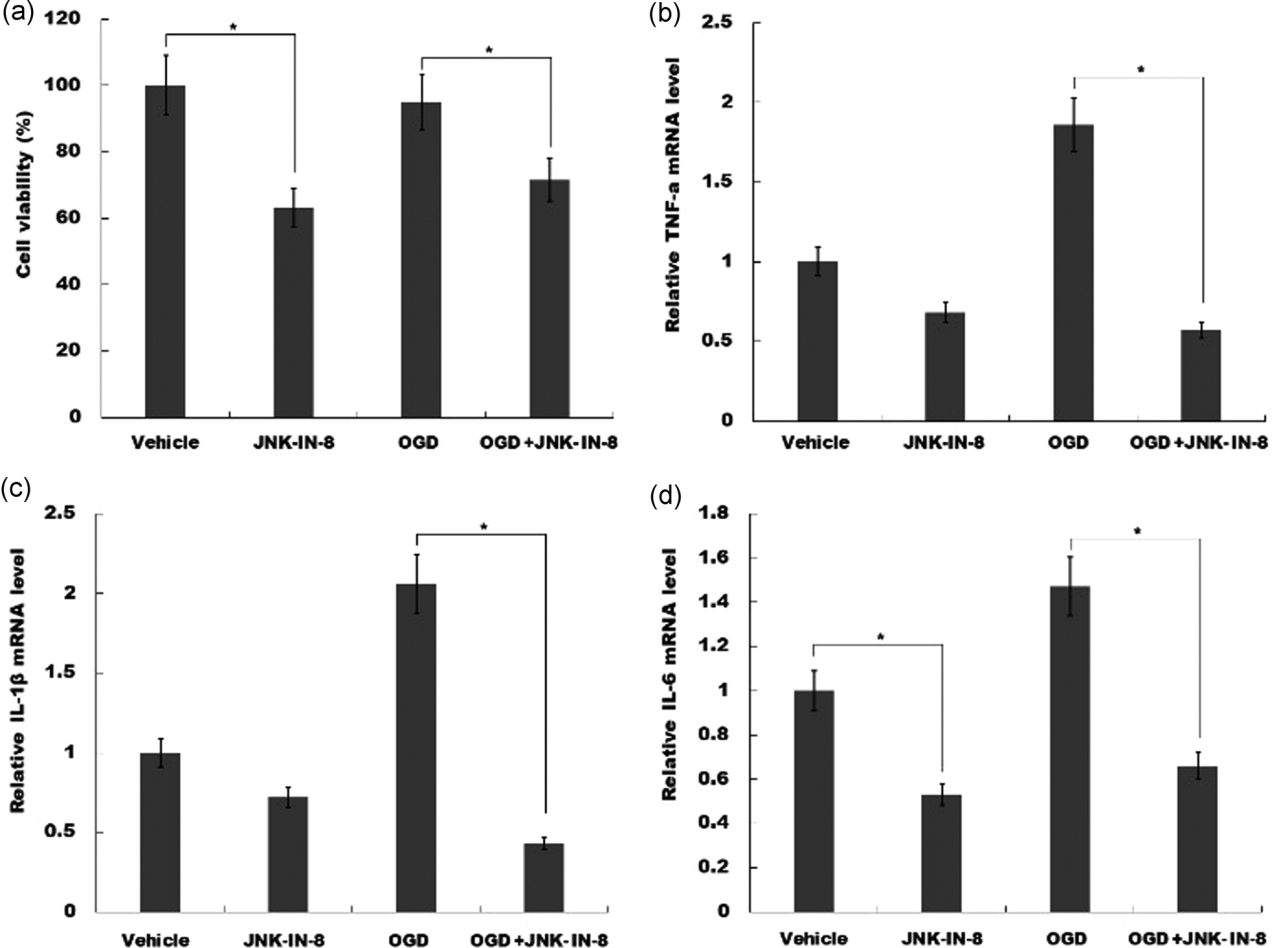
JNK‐IN‐8 inhibits microglia activation and the production of proinflammatory cytokines in vitro. (a) JNK‐IN‐8 treatment (10 mM) inhibited BV2 microglia viability determined by Cell Counting Kit‐8 kit. (b–d) JNK‐IN‐8 treatment (10 mM) reduced the mRNA levels of (b)TNF‐α, (c) IL‐1β, and (d) IL‐6, in BV2 cells determined by quantitative real‐time PCR, BV2 cells under normoxia with OGD groups, BV2 cells subjected to oxygen and glucose deprivation. IL, interleukin; JNK, c‐Jun N‐terminal kinase; mRNA, messenger RNA; OGD, oxygen and glucose deprivation; PCR, polymerase chain reaction; TNF‐α, tumor necrosis factor‐α. *p < .05

To verify JNK‐IN‐8 role on microglia activation in vitro, BV2 microglial cells activation on normoxia or oxygen and glucose deprivation (OGD) with or without JNK‐IN‐8 treatment were assessed by cell counting kit‐8 analysis. As shown in Figure 4a, cell activity of BV2 microglia cells was significantly decreased with JNK‐IN‐8 treatment under normoxia or OGD and indicated JNK‐IN‐8 suppressed the activation of BV2 microglial cells.

We then assessed JNK‐IN‐8 role on proinflammatory cytokines production in vitro. OGD cellular models were used and then BV2 cells were treated with JNK‐IN‐8. Figure 4b–d showed that JNK‐IN‐8 treatment inhibited TNF‐α, IL‐1β, and IL‐6 level under normoxia. Furthermore, these proinflammatory cytokines expression level in BV2 microglia cells with or without JNK‐IN‐8 treatment on OGD was assayed using qPCR. As shown in Figure 4b–d, TNF‐α, IL‐1β, and IL‐6 expression levels were increased under OGD, but JNK‐IN‐8 treatment attenuated OGD‐induced upregulation of TNF‐α, IL‐1β, and IL‐6 expression. These results suggested that JNK‐IN‐8 treatment promotes functional recovery by suppressing JNK and NF‐κB signaling, and attenuating microglial activation and proinflammatory response.

## 4 DISCUSSION

Here, we determined the JNK‐IN‐8 potential effects on protecting against cerebral ischemia injury in a MCAO rat model, and confirm JNK‐IN‐8 precise role in regulations on stroke. The current data demonstrate that (a) JNK‐IN‐8 inhibits the activation of JNK‐NK‐κB pathway, (b) JNK‐IN‐8 inhibits microglia activation in vivo after ischemic stroke, (c) JNK‐IN‐8 inhibits microglia activation and proinflammatory cytokines production in vitro, (d) JNK‐IN‐8 improves functional recovery after stroke. These present data show a potential role of JNK‐IN‐8 in regulating neuroinflammation and could provide a new opportunity for the treatment of ischemic stroke.

Inflammation after ischemia can lead to the occurrence of nerve injury and cerebral infarction (Zhou et al., 2011). However, the progress in appropriate treatments aimed at reducing neuroinflammation remains limited. The JNK, as a MAPK member, regulates various cell functions, such as proliferation, apoptosis, and differentiation (Chen et al., 2018). JNK signaling is reportedly associated with microglia activation and neuroinflammation (Manning & Davis, 2003). Additionally, JNK mediates NF‐κB signaling activation, which is a major upstream modulator for proinflammatory cytokines in microglia (Jiang et al., 2017; Simmons et al., 2016). Therefore, studies on novel therapeutic targets neuroinflammation and neuropathic pain have considered JNK as a promising candidate.

Various JNK‐related synthetic inhibitors have been reported in cerebral ischemia/reperfusion injury, such as micromolecules SP600125 and IQ‐1S. Guan et al. (2006) reported that SP600125 treatment inhibits JNK activation and provides neuroprotection in ischemia/reperfusion via inhibiting neuronal apoptosis. IQ‐1S releases nitric oxide in the course of redox biological transformation process and improves the results of stroke in a cerebral reperfusion mouse model (Atochin et al., 2016). Noteworthily, different JNK inhibitor exerts diverse physiological properties because of targeting different JNK kinds. Thus, it is still a crucial step of further look for selective JNK inhibitors.

In this study, JNK‐IN‐8 role in regulating neuroinflammation and neurological function after stroke was investigated. JNK‐IN‐8 treatment inhibits the activation of stroke‐induced microglia in vivo and in vitro. TNF‐α, IL‐1β, and IL‐6 levels are upregulated under OGD, but JNK‐IN‐8 treatment attenuates OGD‐induced TNF‐α, IL‐1β, and IL‐6 level upregulation. These results suggest that JNK‐IN‐8 treatment contributes to attenuate microglia activation and proinflammatory response. As expected, JNK‐IN‐8 significantly inhibits brain p‐JNK level compared with the vehicle, indicating ischemia‐induced JNK activation was inhibited by JNK‐IN‐8. Furthermore, we demonstrated that JNK‐IN‐8 reduces the activation of NF‐κB signaling. More important, MCAO rats treated with JNK‐IN‐8 exerts a significant improvement in spatial learning and sensory‐motor function recovery were measured through the FFT and mNSS test. Taken together, this study suggests an interesting prospect to target JNK‐IN‐8 as a potent therapy for ischemic stroke.

## FUNDING INFORMATION

The project was supported by the National Natural Science Foundation of China (No. 81774109, No. 81603685, No. 81704180), Zhejiang Provincial Department of Education (No. Y201839270), and Wenzhou Municipal Science and technology Bureau (No. Y20170023, No. Y20170141).

## CONFLICT OF INTERESTS

The authors declare that there are no conflict of interests.

## AUTHOR CONTRIBUTIONS

H. T., O. D., D. J., and S. L. were involved in designing research route and conducting related work, collecting and analyzing data, and writing the manuscript. J. T. and Y. L. helped to draft the manuscript. W. G. was involved in the idea and design of the study, and eventually approved the submitted version. All authors declare that there is no competing interest.

## ETHICS STATEMENT

Animal experiments were approved by the Committee for Animal Experiments at Wenzhou Medical University.

## Supporting information

Supplementary information
