# Supplementary material for: JNK‐IN‐8, a c‐Jun N‐terminal kinase inhibitor, improves functional recovery through suppressing neuroinflammation in ischemic stroke
Source: J Cell Physiol. 2019 Sep 20;235(3):2792–9. doi: 10.1002/jcp.29183 (PMC6916328; doi:10.1002/jcp.29183)
Supplement: Supplementary file 1 — Supplementary information [file JCP-235-2792-s001.docx]

**Supporting** **Table.S1. Primer Sequence in the study.**

| TNF-a  IL-1β  IL-6  β-actin | Primer S  Primer A  Primer S  Primer A  Primer S  Primer A  Primer S  Primer A | CCAGACCCTCACACTCAGATCA  GTAGACAAGGTACAACCCATCGG  TCGCAGCAGCACATCAACAAG  GAAGGTCCACGGGAAAGACACA  TTCACAGAGGATACCACCCACAA  AAACGGAACTCCAGAAGACCAGA  AGATTACTGCCCTGGCTCCTAGC  CCGGACTCATCGTACTCCTGCT |
| --- | --- | --- |
